# Optimization of Communication Signal Adversarial Examples by Selectively Preserving Low-Frequency Components of Perturbations

**DOI:** 10.3390/s24227110

**Published:** 2024-11-05

**Authors:** Yi Zhang, Lulu Wang, Xiaolei Wang, Dianxi Shi, Jiajun Bai

**Affiliations:** 1 Intelligent Game and Decision Lab, Beijing 100071, China; 2 China Special Police College, Beijing 102211, China

**Keywords:** communication signal, adversarial examples, low-frequency, perturbations

## Abstract

Achieving high attack success rate (ASR) with minimal perturbed distortion has consistently been a prominent and challenging research topic in the field of adversarial examples. In this paper, a novel method to optimize communication signal adversarial examples is proposed by focusing on low-frequency components of perturbations (LFCP). Observations on model attention towards DCT coefficients reveal the crucial role of LFCP within adversarial examples in altering the model’s predictions. As a result, selectively preserving LFCP is established as the fundamental concept of the optimization strategy. By utilizing the binary search algorithm, which considers the inconsistency in the model’s predictions as a constraint, LFCP can be effectively identified, and the aim of minimizing perturbed distortion while maintaining ASR can be achieved. Experimental results conducted on a publicly available dataset, six adversarial attacks and two DNN models, indicate that the proposed method not only significantly minimizes perturbed distortion for FGSM, BIM, PGD, and MI-FGSM but also achieves a modest improvement in ASR. Notably, even for DeepFool and BS-FGM, which introduce small perturbations and exhibit high ASRs, the proposed method can still deliver feasible performance.

## 1. Introduction

Adversarial examples are generated by adding carefully crafted subtle perturbations to original samples, causing deep neural network (DNN) models to make incorrect predictions while being imperceptible to human observers. Adversarial attacks, aimed at generating adversarial examples, have become a prevalent and crucial approach in combating artificial intelligence systems at present. With the emergence of DNN models’ empowering effects in various fields, research on adversarial examples has garnered widespread attention in academia and industry [1,2,3,4]. Exploring this area helps to reveal the weaknesses of DNN models, thus driving improvements in the robustness and security of such models, ultimately enhancing the reliability and stability of artificial intelligence systems.

The concept of adversarial examples was initially introduced for images in computer vision (CV) by Szegedy et al. [5]. The limited memory broyden-fletcher-goldfarb-shanno (L-BFGS) algorithm was employed to transform the generation of adversarial examples into a box-constrained formulation, with the $\ell_{2}$ norm of perturbations and the loss of the targeted label serving as the objective function. Subsequently, Goodfellow et al. [6] proposed the fast gradient sign method (FGSM), which leverages the gradient sign information of the loss function to design perturbation. This breakthrough laid the foundation for the flourishing development of adversarial examples. Since then, significant progress has been made in the research on adversarial examples, with researchers proposing a plethora of adversarial attacks based on diverse prior knowledge and iterative optimization approaches [7,8,9,10,11]. Attack success rate (ASR) and perturbed distortion are typically employed to evaluate adversarial examples [11,12,13]. The former metric is reflected by the percentage of adversarial examples that successfully deceive DNN models, while the latter has traditionally been quantified using data-wise differences, such as the $\ell_{p}$ norm, mean square error (MSE) [12], and root mean square error (RMSE, as utilized in this paper) [7,13]. These two metrics highlight the effectiveness and stealthiness of generated adversarial examples, respectively. It is quite natural that achieving high ASR with minimal perturbed distortion has consistently been a prominent and challenging research topic.

Adversarial examples pose an inherent security concern for DNN models, extending beyond CV into various application fields. Within signal processing, Sadeghi et al. [14] were pioneers in exploring communication signal adversarial examples. They utilized the gradient information of the signal under different labels and employed the binary search algorithm to discover minimal perturbation capable of altering the model’s predictions. The proposed adversarial attack, known as the binary search fast gradient method (BS-FGM), effectively showcased the susceptibility of DNN models used in automatic modulation recognition to adversarial examples. Bair et al. [15] examined and analyzed the performance and limitations of two popular adversarial attacks, namely FGSM and moment iterative fast gradient sign method (MI-FGSM) [16], in signal frequency machine learning. Similarly, Flowers et al. [17] assessed the effectiveness of FGSM using a personalized evaluation methodology. Their findings concluded that FGSM surpassed Gaussian noise and proved suitable for over-the-air attacks, while also offering valuable defense recommendations. Lin et al. [18] conducted comprehensive experiments to validate the suitability of four widely-used adversarial attacks on signals: FGSM, basic iterative method (BIM) [19], projected gradient descent (PGD) [20], and MI-FGSM. The results demonstrated the superior performance of the latter three iterative adversarial attacks compared to FGSM. Additionally, Zhao et al. [21] transformed signals into contour stellar images and experimentally confirmed the efficacy of the aforementioned four adversarial attacks. Collectively, these studies significantly contribute to the understanding of communication signal adversarial examples and provide insights into the performance and characteristics of different adversarial attacks.

Currently, most adversarial attacks on communication signals are superficial adaptations of accomplishments in CV. Although they have shown promising ASR outcomes, there is a lack of comprehensive investigation into perturbed distortion. This paper focuses on the optimization of communication signal adversarial examples with the aim of minimizing perturbed distortion while maintaining ASR. Through the observations in discrete cosine transform (DCT) domain, it has been discovered that DNN models tend to allocate high attention to low-frequency components (i.e., DCT coefficients) of communication signals. Consequently, it is believed that low-frequency components of perturbations (LFCP) within adversarial examples play a crucial role in altering the model’s predictions. Motivated by this insight, an optimization method that selectively preserves LFCP for communication signal adversarial examples is proposed. The proposed method employs the binary search algorithm to determine the optimal index threshold that effectively identifies LFCP, taking advantage of the positive correlation between the corresponding frequencies of DCT coefficients and their indices. Furthermore, the inconsistency in the model’s predictions between original samples and optimized examples is considered a constraint during the search process. These ensure that perturbed distortion is minimized as much as possible without compromising ASR. The main innovations of the proposed method include the optimization strategy that selectively preserves LFCP and its specific implementation based on the binary search algorithm.

The subsequent sections of this paper are organized as follows: Section 2 presents a review of typical adversarial attacks that will be utilized in the experiments, along with an introduction to DCT transformation and an exploration into the distributions of perturbations in the DCT domain. The optimization strategy and optimization algorithm are extensively described in Section 3. Subsequently, Section 4 presents the experimental results, which provide empirical evidence to support the proposed method. Finally, Section 5 concludes the paper and delves into future research prospects.

## 2. Related Work

This section presents the principles of six typical adversarial attacks that can be applied to communication signals, along with their symbolic representations to enhance comprehension. Moreover, the principle and properties of DCT transformation are introduced, and the distributions of perturbations in the DCT domain are also explored.

### 2.1. Adversarial Attacks

Given a DNN model, $f_{\theta }\left(\cdot \right)$ with parameters θ, an original sample x with ground-truth label $y=f_{\theta }\left(x\right)$ and data space R, adversarial example $x^{\prime }$ can be generated by solving the following optimization problem:

(1) $$\min_{\eta }{∥\eta ∥}_{p}\;\;\;s.t.\;\;f_{\theta }\left(\underset{x^{\prime }}{\underset{︸}{x+\eta }}\right)=y^{\prime }\neq y\;\mathrm{and}\;x^{\prime }\in R$$

where η is the perturbation which is added to x, ${∥\eta ∥}_{p}$ represents the $\ell_{p}$ norm (p∈[0,2,∞]) of η. Equation (1) is to find the minimal η that is subject to two constraints: (a) x and $x^{\prime }$ have inconsistent predicted labels; (b) $x^{\prime }$ shares the same data space as x. In practice, a direct solution to the aforementioned optimization problem is not feasible. Therefore, researchers have developed several approaches to enable effective generation of adversarial examples. A succinct overview of six typical adversarial attacks is as follows:

FGSM is a gradient-based one-step attack, which utilizes the gradient sign of the loss function to design perturbation. The generation process can be described as follows:

(2) $$x^{\prime }=x+\varepsilon \cdot sign\left(\nabla_{x}J\left(x,y,\theta \right)\right)$$

where ε is the parameter that controls perturbation, $\nabla_{x}J\left(x,y,\theta \right)$ is the gradient of loss function $J\left(\cdot \right)$ with respect to sample x and ground-truth label *y*, and $sign\left(\cdot \right)$ is the sign function.

BIM is an advanced version of FGSM, which iteratively updates adversarial examples by leveraging the gradient sign of the loss function, resulting in more subtle perturbation. The generation process can be described as follows:

(3) $$\begin{array}{cc} \; & x_{0}^{\prime }=x\\ \; & x_{k+1}^{\prime }=clip_{x,\varepsilon }\left\{x_{k}^{\prime }+\alpha \cdot sign\left(\nabla_{x_{k}^{\prime }}J\left(x_{k}^{\prime },y,\theta \right)\right)\right\} \end{array}$$

where $x_{k}^{\prime }$ is the sample at the *k*-th iteration, $clip_{x,\varepsilon }\left\{\cdot \right\}$ is the operation that clips the input to a specified range $\left[x-\varepsilon ,x+\varepsilon \right]$, ε, and α are parameters that respectively control the overall perturbation and the perturbation added at each iteration.

PGD is an extension of BIM that incorporates random noise during initialization to enhance exploration of the perturbation space, thereby generating more diverse and robust adversarial examples. The generation process can be described as follows:

(4) $$\begin{array}{cc} \; & x_{0}^{\prime }=x+\xi \\ \; & x_{k+1}^{\prime }=\prod_{B\left(x,\varepsilon \right)}\left\{x_{k}^{\prime }+\alpha \cdot sign\left(\nabla_{x_{k}^{\prime }}J\left(x_{k}^{\prime },y,\theta \right)\right)\right\} \end{array}$$

where ξ∼U(−ε,ε) is a uniform random noise, $\prod_{B(x,\varepsilon )}\left\{\cdot \right\}$ is the projection operation that constrains the input within the neighborhood space of x, denoted as B(x,ε).

**MI-FGSM** is similar to BIM, which stabilizes the direction of perturbation and prevents falling into local extremes by introducing a momentum term during iterations. The generation process can be described as follows:

(5) $$\begin{array}{cc} \; & x_{0}^{\prime }=x,m_{0}=0\\ \; & m_{k+1}=\mu \cdot m_{k}+\frac{\nabla_{x_{k}^{\prime }}J\left(x_{k}^{\prime },y,\theta \right)}{{∥\nabla_{x_{k}^{\prime }}J\left(x_{k}^{\prime },y,\theta \right)∥}_{1}}\\ \; & x_{k+1}^{\prime }=x_{k}^{\prime }+\alpha \cdot sign\left(m_{k+1}\right) \end{array}$$

where $m_{k}$ is the momentum term at the *k*-th iteration, μ is the momentum decay coefficient, and α is the parameter that controls the perturbation added at each iteration.

DeepFool [22] is also an iterative attack that approximates DNN models as linear decision boundaries, calculating the minimal perturbation required to cross boundary at each iteration. It repeats this process until the attack succeeds or reaches a maximum number of iterations. The generation process can be described as follows:

(6) $$\begin{array}{cc} \; & x_{0}^{\prime }=x\\ \; & l_{k}=\underset{y^{\prime }\neq y}{\arg\min}\frac{\left|L\left(x_{k}^{\prime },y^{\prime },\theta \right)-L\left(x_{k}^{\prime },y,\theta \right)\right|}{{∥\nabla_{x_{k}^{\prime }}L\left(x_{k}^{\prime },y^{\prime },\theta \right)-\nabla_{x_{k}^{\prime }}L\left(x_{k}^{\prime },y,\theta \right)∥}_{2}}=\underset{y^{\prime }\neq y}{\arg\min}\frac{\left|L^{\prime }\left(y^{\prime }\right)\right|}{{∥\omega \left(y^{\prime }\right)∥}_{2}}\\ \; & x_{k+1}^{\prime }=x_{k}^{\prime }+\frac{\left|L^{\prime }\left(l_{k}\right)\right|}{{∥\omega \left(l_{k}\right)∥}_{2}^{2}}\cdot \omega \left(l_{k}\right) \end{array}$$

where $l_{k}$ is the label corresponding to the decision boundary closest to sample $x_{k}^{\prime }$ at the *k*-th iteration, the function L(·) represents DNN model’s logits output, while its gradients with respect to $x_{k}^{\prime }$, label $y^{\prime }$ and $x_{k}^{\prime }$, label *y* are denoted as $\nabla_{x_{k}^{\prime }}L\left(x_{k}^{\prime },y^{\prime },\theta \right)$ and $\nabla_{x_{k}^{\prime }}L\left(x_{k}^{\prime },y,\theta \right)$.

**BS-FGM** is an attack specifically designed for communication signals, which determines the minimal perturbation for each label by employing the binary search algorithm within a predefined search interval along the normalized gradient direction. The final perturbation is derived by selecting the smallest from all the calculated minimal perturbations. The generation process can be described as follows:

(7) $$\begin{array}{cc} \; & r_{y^{\prime }}=\frac{\nabla_{x}J\left(x,y^{\prime },\theta \right)}{{∥\nabla_{x}J\left(x,y^{\prime },\theta \right)∥}_{2}}\\ \; & \varepsilon_{y^{\prime }}=BS\left(\varepsilon_{min},\varepsilon_{max},\varepsilon_{acc};f_{\theta }\left(x-\varepsilon \cdot r_{y^{\prime }}\right)\neq y\right)\\ \; & y^{*}=\underset{y^{\prime }\neq y}{\arg\min}\left(\varepsilon_{y^{\prime }}\right)\\ \; & x^{\prime }=x-\varepsilon_{y^{*}}\cdot r_{y^{*}} \end{array}$$

where $r_{y^{\prime }}$ is the normalized gradient of loss function J(·) with respect to sample *x* and label $y^{\prime }$. The binary search algorithm BS(·) iterates within the interval $\left[\varepsilon_{min},\varepsilon_{max}\right]$, searching for the minimal scale factor $\varepsilon_{y^{\prime }}$ that satisfies $f_{\theta }\left(x-\varepsilon \cdot r_{y^{\prime }}\right)\neq y$ with a search precision of $\varepsilon_{acc}$ for label $y^{\prime }$. The final perturbation is determined by the smallest scale factor $\varepsilon_{y^{*}}$.

### 2.2. DCT Transformation

DCT transformation serves as a widely employed approach for time-frequency conversion in signal processing. This approach decomposes signals into cosine functions characterized by varying amplitudes and frequencies. The resulting DCT coefficients directly represent the amplitudes of these cosine functions with a positive correlation between their corresponding frequencies and indices. It exhibits two significant properties: Firstly, the energy concentration property centralizes the majority energy of the signal within low-frequency DCT coefficients after transformation, enabling efficient signal compression and denoising that relies on these coefficients. Secondly, the linear reversible property facilitates efficient computations and accurate signal reconstruction across various signal processing applications.

In DNN-based signal processing, it is a common practice to represent communication signals as two-channel inputs by utilizing their in-phase (I) and quadrature (Q) components. This representation, referred to as IQ signal(s), will be employed throughout the subsequent sections. Given an adversarial example $x^{\prime }$ generated based on IQ signal x and perturbation η, all of which belong to the data space $R^{N\times 2}$, with *N* denoting the sampling length and ‘2’ indicating the complex nature of I and Q components. Based on the linear reversible property of DCT, their DCT coefficients, denoted as $d^{\prime }$, d, and $d^{\eta }$, exhibit the following transformation relationship:

(8) $$\begin{array}{cc} d^{\prime } & =D\left(x^{\prime }\right)=D\left(x+\eta \right)\\ \; & =D\left(x\right)+D\left(\eta \right)\\ \; & =d+d^{\eta } \end{array}$$

where D(·) denotes the DCT transformation function, which is applied independently to each channel of IQ signal.

**Figure 1:**
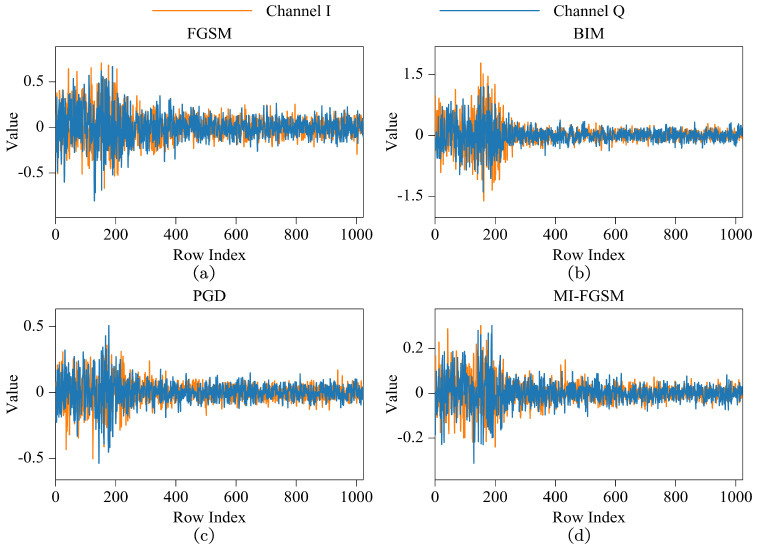

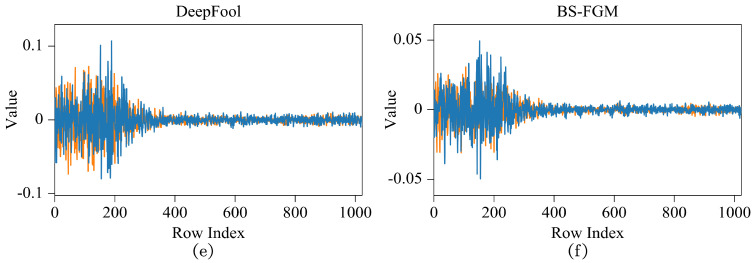
The DCT coefficients of various perturbations. Subplots (**a**–**f**) illustrate specific types of perturbations generated by FGSM, BIM, PGD, MI-FGSM, DeepFool, and BS-FGM, respectively.

Frequency transformations, including DCT, provide valuable insights into the inherent characteristics of adversarial examples in CV, enabling both the enhancement of adversarial attack transferability [23,24] and the development of robust defenses against adversarial attacks [25,26]. To explore the characteristics of perturbations in the DCT domain, an IQ signal from RadioML2018.01A (https://www.deepsig.ai/datasets/, accessed on 14 May 2024) was selected as the original sample. Six distinct types of perturbations were generated using adversarial attacks described in the previous subsection. The DCT coefficients of various perturbations are illustrated in Figure 1. The horizontal axis represents the row index of DCT coefficients, while the vertical axis indicates their values. It is evident that perturbations are widely distributed across the DCT domain in all subplots, with a primary concentration observed in low-frequency DCT coefficients. This phenomenon may be attributed to the energy concentration property associated with DCT transformation and the nature of adversarial attacks, which introduce perturbations in the original domain without imposing constraints in the frequency domain.

## 3. Methodology

This section first analyzes model attention towards DCT coefficients, with the aim of providing valuable insights to guide the design of optimization strategy. Following this, the optimization strategy based on these insights is presented, accompanied by preliminary experimental results to validate its effectiveness. Finally, a comprehensive description of the optimization algorithm essential for implementing the proposed strategy is provided.

### 3.1. Model Attention Towards DCT Coefficients

Similar to the original domain data points within the input sample (e.g., pixels within an image), the frequency domain data points, such as DCT coefficients, also demonstrate varying levels of model attention. These variations yield insights into their diverse contributions to the model’s predictions. Due to the energy property characteristics of DCT transformation, the major energy of a communication signal is mainly concentrated in low-frequency DCT coefficients. Therefore, DNN models should allocate high attention to these coefficients in order to effectively extract the fundamental features and crucial data embedded in the signal, thereby ensuring the performance of prediction.

**Figure 2:**
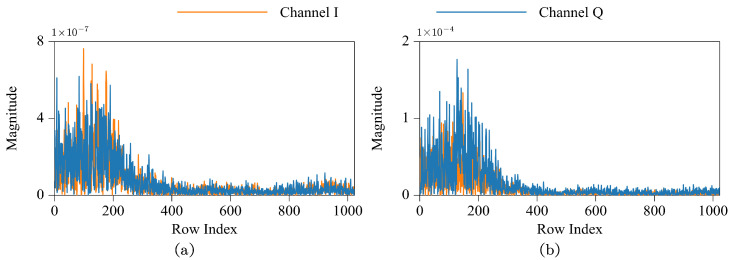

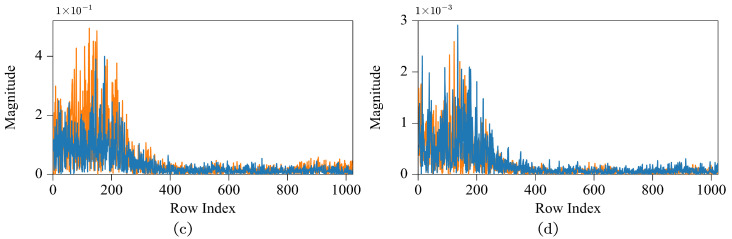
The gradient magnitudes of DCT coefficients under CNN model. Subplots (**a**–**d**) correspond to distinct IQ signals from RadioML2018.01A.

Model attention towards DCT coefficients can be approximately quantified through data points’ gradients [23,27]. A larger gradient magnitude generally indicates a greater level of model attention towards that particular DCT coefficient, implying a more significant contribution to the model’s predictions, and vice versa. In order to validate the aforementioned inference, an investigation on the gradient magnitudes of DCT coefficients was conducted under CNN model (see Section 4.1.1). Figure 2 illustrates four subplots, each corresponding to an IQ signal from RadioML2018.01A. The horizontal axis represents the row index of DCT coefficients, while the vertical axis represents the gradient magnitude. Notably, all subplots exhibit similar patterns: the indices of DCT coefficients with larger gradient magnitudes predominantly concentrate on the left region of the horizontal axis, spanning both I and Q components, which correspond to low-frequency DCT coefficients. This observation suggests a higher level of model attention towards low-frequency DCT coefficients, which significantly contribute to the model’s predictions.

### 3.2. Optimization Strategy

The linear combination relationship presented in Equation (8) demonstrates a one-to-one correspondence among original samples, perturbations, and adversarial examples in the DCT domain. Specifically, low-frequency components of original samples, when combined with those of perturbations, yield low-frequency components of adversarial examples, and the same applies to high-frequency components. Based on the principles of adversarial attacks outlined in Section 2.1, the inconsistency in the model’s predictions between original samples and adversarial examples primarily arises from perturbations, which can be attributed to the various frequency components of perturbations from the perspective of the DCT domain. Given that high model attention is directed towards low-frequency components of original samples, the added low-frequency components of perturbations (LFCP) should play a significant role in altering the model’s predictions. Therefore, selectively preserving LFCP has the potential to minimize perturbed distortion while still maintaining the incorrect model’s prediction, representing the fundamental idea of the optimization strategy.

To implement the proposed optimization strategy, it is crucial to identify LFCP. One practical approach involves establishing a criterion for distinguishing between high- and low-frequency components. While there is no strict criterion, an index threshold approach can be utilized, taking advantage of the positive correlation between the corresponding frequencies of DCT coefficients and their indices. By setting the index threshold as n∈[0,N−1], LFCP can be defined as the collection of DCT coefficients whose indices fall within the set $\Delta =\left\{\left.\left(u,v\right)\right|0⩽u⩽n,0⩽v⩽1\right\}$. An optimized example, denoted as $x^{*}=\mathrm{opt}\left(x,x^{\prime },n\right)$, can be obtained by applying the optimization strategy as follows:

(9) $$\begin{array}{cc} \; & \eta =x^{\prime }-x\\ \; & d^{\eta }=D\left(\eta \right)\\ \; & d_{low}^{\eta }=d^{\eta }⊙m\\ \; & x^{*}=x+D_{I}\left(d_{low}^{\eta }\right) \end{array}$$

In Equation (9), LFCP, denoted as $d_{low}^{\eta }$, is derived through Hadamard operation, symbolized by ⊙. This process involves DCT coefficients of perturbation $d^{\eta }$ and masking matrix m. $D_{I}\left(\cdot \right)$ denotes the inverse function of DCT transformation. The element $m_{uv}$ at index (u,v) in m is defined as:

(10) $$m_{uv}=\left\{\begin{array}{c} 1,\;if\;\;0⩽u⩽n,0⩽v⩽1\\ 0,\;otherwise \end{array}\right.$$

The given definition implies a close connection between m and Δ. In particular, elements in m corresponding to indices in Δ are set to 1, whereas the rest are set to 0. Hence, Equation (10) signifies the preservation of LFCP while discarding high-frequency components of perturbations.

**Figure 3:**
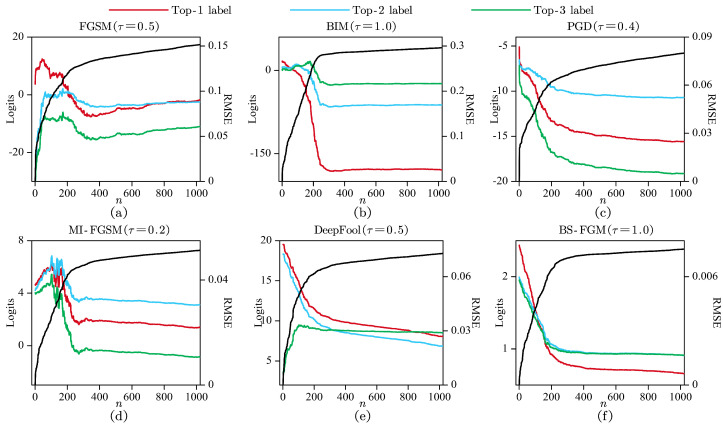
CNN model’s logits (colored lines) and RMSEs (black lines) of optimized examples with varying index thresholds *n*. Subplots (**a**–**f**) correspond to adversarial examples generated by FGSM, BIM, PGD, MI-FGSM, DeepFool, and BS-FGM, respectively.

In order to provide initial validation for the proposed optimized strategy, an investigation was conducted on the model’s logits and RMSEs of optimized examples. This investigation involved applying the strategy to adversarial examples with varying index thresholds *n*. The findings are illustrated in Figure 3, where each subplot corresponds to an adversarial example generated by a specific adversarial attack with an IQ signal from RadioML2018.01A under CNN model (see Section 4.1.1). The horizontal axis represents index threshold *n*, the left vertical axis represents logits for different labels (colored lines), and the right vertical axis represents RMSE (black lines). For clarity, only the logits associated with Top-1, Top-2, and Top-3 labels that obtained the largest scores are included in the illustration. It can be observed that:

(1) In all subplots, when dealing with small values of *n*, the logits associated with Top-1, Top-2, and Top-3 labels exhibit considerable variation. However, as *n* increases, these logits become more stable. Notably, for small values of *n*, the logits associated with Top-1 are already lower than those associated with Top-2 or Top-3 labels, indicating that LFCP is sufficient to alter the model’s predictions. Furthermore, as *n* increases, RMSE becomes larger but more stable and less sensitive to further changes, indicating that there exists a non-linear and proportional relationship between RMSE and *n*;

(2) In subplot (a), as *n* increases, the label with the largest logits value successively transitions from Top-1 label to Top-2 label and back to Top-1 label, indicating that excessively large perturbations may result in unsuccessful attacks.

In summary, the observations above suggest that the optimization strategy, which focuses on LFCP within adversarial examples, can minimize perturbed distortion to some extent. Moreover, there is potential for this strategy to further improve ASR.

### 3.3. Optimization Algorithm

Building upon the previous discussions, it is evident that index threshold *n* holds a pivotal position in influencing the overall performance of the optimization strategy in practical applications. When a small value of *n* is utilized, the resulting optimized example may exhibit a low RMSE but maintain a consistent label compared to the original sample, thereby compromising ASR. Conversely, employing a large value of *n* may result in an optimized example with inconsistent label but high RMSE, thereby compromising perturbed distortion. Both of these scenarios ultimately undermine the effectiveness of optimization. To achieve the optimization objective, it is essential to identify the optimal index threshold $n^{*}$ by prioritizing ASR while simultaneously minimizing perturbed distortion. Consequently, the optimization problem can be reframed as the identification of $n^{*}$, which is defined as the smallest value that satisfies two constraints concurrently: (1) the model’s predictions for original sample *x* and optimized example $x^{*}$ are inconsistent; (2) $x^{*}$ belongs to the same data space as *x*. To formulate explicitly, the reframed problem can be described as follows:

(11) $$n^{*}=\min n\;\;\;\;\;\;s.t.\;f_{\theta }\left(\underset{x^{*}}{\underset{︸}{\mathrm{opt}(x,x^{\prime },n)}}\right)\neq y\;\;and\;\;x^{*}\in R^{N\times 2}$$

This redefinition enables the simultaneous consideration of the inconsistency in the model’s predictions and data space alignment, ensuring a delicate balance between maintaining ASR and minimizing perturbed distortion.

Due to the unique properties of different adversarial examples and the complexity of adversarial attacks, it is not feasible to establish a fixed value for $n^{*}$. Meanwhile, rigorous calculations are unable to identify $n^{*}$, and conducting an exhaustive search for all potential values of *n* demands substantial computational resources and time, rendering it impractical for practical applications. To overcome these challenges, the binary search algorithm is employed, and the optimization algorithm (Algorithm 1) can be summarized as follows:
**Algorithm 1** The Proposed Optimization Algorithm**Inputs:** Original sample x labeled with *y*; Adversarial example $x^{\prime }$ labeled with $y^{\prime }$; DNN model $f_{\theta }\left(\cdot \right)$ **Output:** Optimized example $x^{*}$
  1:**Initialization**: $\lambda_{min}\leftarrow 0$, $\lambda_{max}\leftarrow N-1$, $\lambda_{acc}\leftarrow 1$, and $n^{*}\leftarrow N-1$  2:**while**$\;\lambda_{max}-\lambda_{min}>\lambda_{acc}\;$**do**  3:    $\lambda_{ave}\leftarrow \frac{\left(\lambda_{max}+\lambda_{min}\right)\;\;}{\;\;2}$  4:    $n\leftarrow \lambda_{ave}$  5:    $x^{*}\leftarrow \mathrm{opt}\left(x,x^{\prime },n\right)$           According to Equation (9)  6:    **if** $f_{\theta }\left(x^{*}\right)\neq y$ **then**  7:        $\lambda_{max}\leftarrow \lambda_{ave}$  8:        $n^{*}\leftarrow n$  9:    **else**10:        $\lambda_{min}\leftarrow \lambda_{ave}$11:    **end if**12:**end while**13:**Return **$x^{*}=\mathrm{opt}\left(x,x^{\prime },n^{*}\right)$


where $\lambda_{min}$ and $\lambda_{max}$ denote the lower and upper bounds of the search interval, respectively. Additionally, the search precision is determined by the empirical parameter $\lambda_{acc}=1$, which aims to find the best $n^{*}$, thereby minimizing perturbed distortion as much as possible. In the proposed algorithm, the search interval is iteratively narrowed down by the binary search algorithm, which divides it into two sub-intervals and examines the middle point. By comparing the model’s predictions for the original sample and optimized example at the middle point, it can be determined whether to continue the search in the lower or upper sub-interval.

## 4. Experiments

This section commences by detailing the experimental setup, which includes DNN models utilized as well as the construction process for original sample, adversarial example, and optimized example sets. Subsequently, a comprehensive analysis of the experimental investigations on ASR and perturbed distortion is presented. All experiments were implemented using Pytorch and the NVIDIA RTX 3090 GPU platform.

### 4.1. Experimental Setup

#### 4.1.1. DNN Models

**Figure 4:**
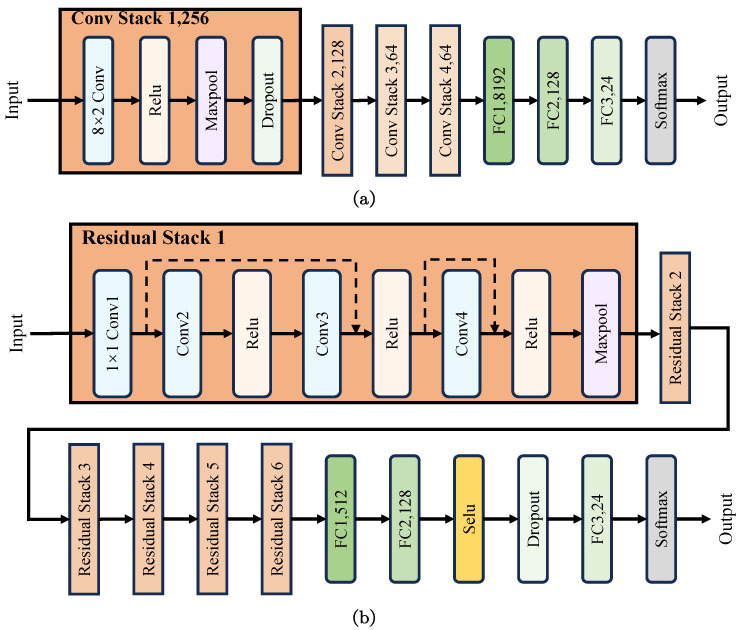
The structures of CNN (**a**) and ResNet (**b**) models.

**Model Structure:** The effectiveness of CNN and ResNet in DNN-based automatic modulation recognition has been established in existing research [28]. Therefore, these two models were selected for experimentation. Figure 4 illustrates the structures of both models. CNN [29] employs four convolutional modules (Conv Stack) with the same structure, and the convolutional layers within each module share the same kernel size. In ResNet [30], six residual modules (Residual Stack) are utilized with the same structure. However, in Residual Stack 1, the kernel sizes of conv2, conv3, and conv4 are 3 × 2, while that of the maxpooling layer is 2 × 2. In the subsequent Residual Stacks, the kernel sizes of these layers are 3 × 1 and 2 × 1, respectively.

**Figure 5:**
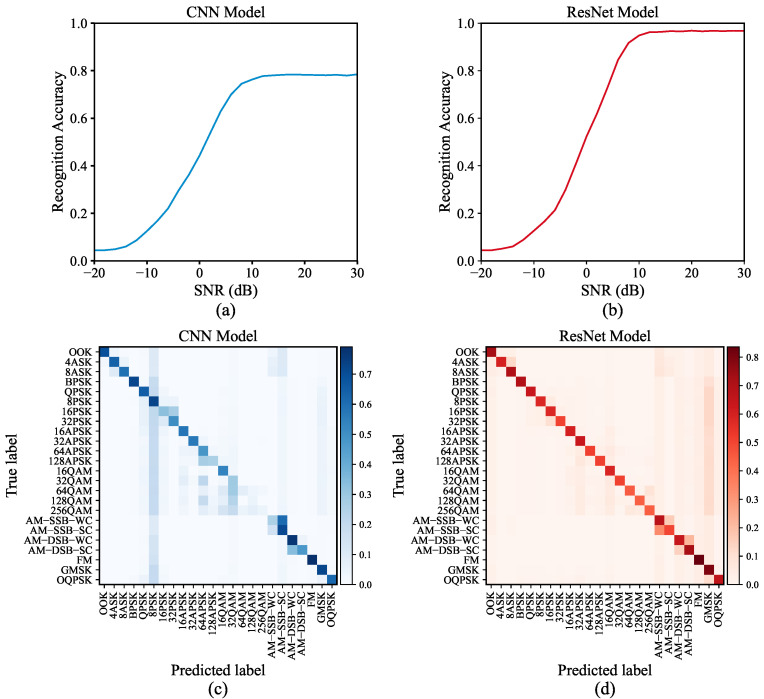
The performance of CNN and ResNet. Subplots (**a**,**b**) present the recognition accuracy of two models across different SNRs, while subplots (**c**,**d**) depict their confusion matrices, respectively.

**Model Training:** RadioML2018.01A [31], an publicly available dataset with 24 modulations and 26 signal-to-noise ratios (SNRs), was utilized as the foundational dataset. This synthetic dataset consists of 2,555,904 IQ signals, each represented by a 1024 × 2 vector with a sampling length of 1024. To train models, the dataset was randomly partitioned into training and test sets using a 7:3 split ratio. During the training process, cross-entropy loss function and Adam optimizer were employed. Meanwhile, both models followed a widely used approach in DNN-based signal processing, treating IQ signals as two-channel inputs. The performance of trained models on the test sets is illustrated in Figure 5.

#### 4.1.2. Original Sample, Adversarial Example, and Optimized Example Sets

The purpose of constructing original sample, adversarial example, and optimized example sets is to enhance the representativeness and robustness of the experiments. By utilizing these sets, the effectiveness and generalizability of the proposed optimization algorithm can be accurately evaluated, leading to more reliable results.

**Original Sample Sets:** In order to proceed adversarial example and optimized example sets, it is necessary to introduce the composition of original sample sets. For each modulation within the test set, 1000 samples that model can make correct classifications were randomly selected, resulting in an original sample set comprising 24 × 1000 = 24,000 samples for each model. Consequently, there are two original sample sets in the experiments. It is worth noting that due to these two models’ limited ability in low SNRs, equal random selection based on modulation rather than SNR was conducted to ensure the diversity of original sample sets.

**Adversarial Example Sets:** With original sample sets in place, a total of six adversarial attacks, as introduced in Section 2.1, were employed to generate adversarial example sets. FGSM, BIM, PGD, MI-FGSM, and DeepFool were implemented using codes provided by Torchattacks (https://github.com/Harry24k/adversarial-attacks-pytorch, accessed on 10 August 2024) [32], while BS-FGM was adapted from the author’s publicly available TensorFlow code (https://github.com/meysamsadeghi/Security-and-Robustness-of-Deep-Learning-in-Wireless-Communication-Systems, accessed on 20 August 2024). The specific parameter configurations for these attacks can be found in Table 1. To facilitate the comparison and analysis of experimental results, the parameters ε in FGSM, BIM, PGD, and MI-FGSM, steps in DeepFool, and $\varepsilon_{max}$ in BS-FGM were defined as functions of the perturbation parameter τ. Meanwhile, to enhance the diversity of adversarial example sets, the range of τ was set to τ∈[0.1,0.2,…,0.9,1.0]. Following the parameter configurations, each model, adversarial attack, and perturbation parameter τ corresponded to an adversarial example set comprising 24,000 examples. Consequently, the total number of adversarial sample sets amounted to 2 × 6 × 10 = 120.

**Optimized Example Sets:** After implementing Algorithm 1 on each example within all adversarial example sets, a collection of 120 corresponding optimized example sets, each containing 24,000 examples, was generated. The parameters of the algorithm were configured as follows: N=1024, corresponding to the sampling length of original samples and adversarial examples.

### 4.2. Experimental Investigations

#### 4.2.1. Comparison of Attack Success Rate

As adversarial examples were generated from original samples that the model can make correct classifications, the ASR of each adversarial or optimized set was equivalent to the model’s misclassification rate on that specific set. In each row of Table 2 (under CNN model) and Table 3 (under ResNet model), ASRs of adversarial example sets ($X^{\prime }$), generated by a specific adversarial attack with varying perturbation parameters τ, are presented alongside the corresponding optimized example sets ($X^{*}$). To facilitate comparison, the higher ASR of the two types of sets with the same τ was highlighted in bold font within each row. The observations based on these two tables are as follows:

(1) As τ increases, adversarial example sets exhibit varying degrees of improvement in ASR. Among the six adversarial attacks, the sets generated by DeepFool and BS-FGM consistently demonstrate advantages, particularly with small τ. By considering the relationship between τ and the parameters of adversarial attacks, it can be concluded that allowing for larger perturbations in FGSM, BIM, PGD, and MI-FGSM contributes to higher ASRs. Meanwhile, ASR can be improved by increasing the number of iterations in DeepFool and enlarging the search interval in BS-FGM. Similarly, there was a consistent relationship between ASR and τ for optimized example sets.

(2) ASRs of optimized example sets consistently exceed or equal to those of the corresponding adversarial example sets in each row of the two tables. Notably, even for adversarial example sets with significantly high (above 99.00%) or notably low (below 50.00%) ASRs, the corresponding optimized example sets can still contribute to a certain degree of improvement. In Table 2, optimized example sets exhibit maximum improvements across various adversarial attacks, with gains of 1.03%, 0.01%, 0.02%, 0.02%, 1.54%, and 0.10%, respectively. Similarly, in Table 3, the corresponding maximum improvements are 2.93%, 0.05%, 0.07%, 0.05%, 2.10%, and 0.17%. Additionally, it should be noted that in approximately half of the cases where ASRs are reported as equal, the optimized example set actually demonstrates a slight advantage, which is indeed attributed to decimal precision limitations. These observations clearly demonstrate that the proposed method performs as expected in ASR and highlight its potential to improve ASR, even though it is not its primary objective.

#### 4.2.2. Comparison of Perturbed Distortion

**Figure 6:**
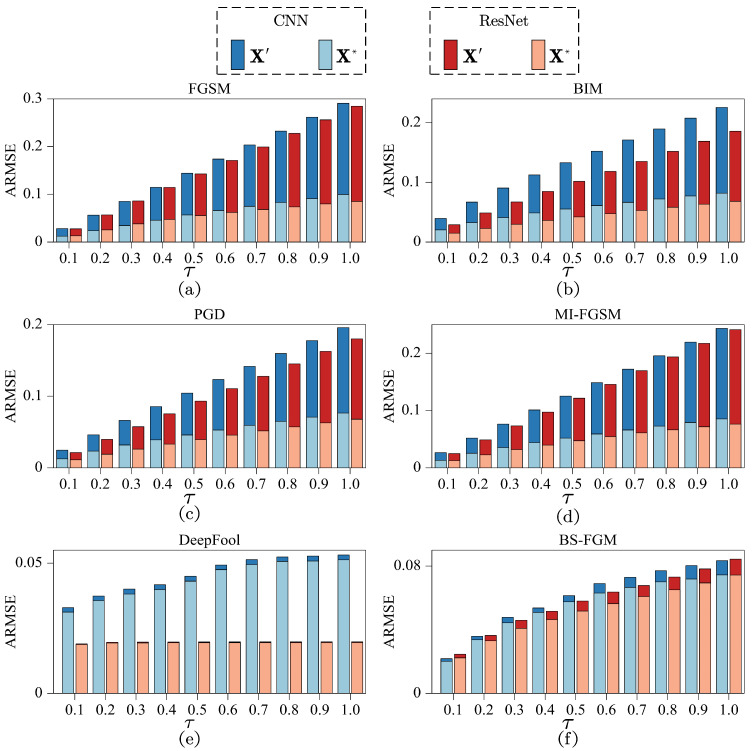
ARMSEs of adversarial example sets ($X^{\prime }$) generated by FGSM (**a**), BIM (**b**), PGD (**c**), MI-FGSM (**d**), DeepFool (**e**), and BS-FGM (**f**) with varying perturbation parameters τ, along with the corresponding optimized example sets ($X^{*}$).

In order to comprehensively and objectively evaluate the performance of the proposed method in minimizing perturbed distortion and enhancing result reliability, a comparison based on the average RMSE (ARMSE) was conducted. ARMSE was obtained by calculating RMSEs of adversarial or optimized examples in each set that exhibited inconsistent model predictions compared to their corresponding original samples. These individual RMSEs were subsequently averaged to derive ARMSE. In each subplot of Figure 6, ARMSEs of adversarial example sets ($X^{\prime }$), generated by a specific adversarial attack with varying perturbation parameters τ, are presented alongside the corresponding optimized example sets ($X^{*}$). The horizontal axis represents perturbation parameter τ, while the vertical axis represents ARMSE. To differentiate between the two types of sets generated under CNN model, dark and light blue bars are employed. Similarly, dark and light red bars are used to signify these two types of sets generated under ResNet model, respectively. It can be observed that:

(1) In subplots (a)–(d), significant minimizations in ARMSE of optimized example sets, represented by light bars, are consistently observed compared to the corresponding adversarial example sets, represented by dark bars. To quantitatively demonstrate this, the average minimizations of optimized example sets across various τ are evaluated. Under CNN model, the achieved average minimizations in these subplots are 61.4%, 57.9%, 55.6%, and 58.4%. Similarly, under ResNet model, the achieved average minimizations are 61.9%, 58.1%, 57.0%, and 60.7%, respectively. Furthermore, as τ increases, both types of sets exhibit an increase in ARMSE. Specifically, adversarial example sets show a more significant increase in ARMSE, demonstrating an approximate linear relationship with τ. In contrast, optimized example sets displays only a weak increase in ARMSE with the increase of τ. Consequently, the minimizations in ARMSE of optimized example sets also increase as τ increases. These observations suggest that the proposed method can effectively minimize the perturbed distortion of adversarial examples generated by FGSM, BIM, PGD, and MI-FGSM. Additionally, it is worth noting that the performance of the proposed method improves as perturbations increases.

(2) In subplots (e) and (f), the less pronounced minimizations in ARMSE are observed. Under CNN model, average minimizations of 4.1% and 8.0% are achieved, while under ResNet model, average minimizations of 1.4% and 10.7% are achieved, respectively. This can be attributed to DeepFool and BS-FGM already dedicating substantial time and computational resources to find optimal perturbations, which limits the optimization potential of adversarial examples generated by these two adversarial attacks. Nonetheless, achieving such minimizations is therefore a noteworthy accomplishment. Meanwhile, DeepFool enhances generation efficiency by implementing an earlystop mechanism that terminates the iteration process upon successful generation. BS-FGM expands the search interval by adjusting the upper bound, resulting in the expanded interval encompassing the interval prior to expansion. As a result, in theory, RMSEs of adversarial examples generated by them would remain unchanged with the increase of τ, and the corresponding optimized examples would also remain unaffected. The slight variations in ARMSE with the increase of τ are mainly attributed to the inclusion of new examples in the statistics. These observations suggest that the proposed method still has certain optimization performance for adversarial examples with small perturbations generated by DeepFool and BS-FGM. Despite the smaller perturbations introduced by these two adversarial attacks compared to the previous four, it can also be concluded that the performance of the proposed method improves as perturbations increases.

## 5. Conclusions

This paper investigates the optimization of communication signal adversarial examples and proposes a method for selectively preserving LFCP that has the most significant impact on altering the model’s predictions. Comparative experiments were not conducted due to the absence of existing methods to optimize communication signal adversarial examples. Nevertheless, the meticulously designed experiments provide compelling evidence of the proposed method’s effectiveness, highlighting its capacity to minimize perturbed distortion and demonstrating its positive impact on ASR. It is important to note that images and audios can also be considered as specific types of signals, and the proposed method is not limited to a particular signal style. Therefore, in future work, we will explore its applicability in optimizing adversarial examples for images and audios. Additionally, apart from DCT, commonly used time-frequency transformation approaches such as discrete fourier transform (DFT) and discrete wavelet transform (DWT) exhibit different properties. Thus, we will investigate the feasibility of employing other transformation approaches for optimizing adversarial examples.

## Figures and Tables

**Table 1:** Parameter configurations of adversarial attacks.

Adversarial Attacks	Parameters
FGSM	ε=τ
BIM	ε=τ, α=ε/5, steps=10
PGD	ε=τ, α=ε/5, steps=10
MI-FGSM	ε=τ, α=ε/5, steps=10, μ=1.0
DeepFool	steps=100·τ, overshoot=0.02
BS-FGM	$\varepsilon_{max}=10\cdot \tau$, $\varepsilon_{min}=0$, $\varepsilon_{acc}=0.1$

**Table 2:** ASRs of adversarial ($X^{\prime }$) and optimized ($X^{*}$) example sets generated under CNN model.

Example Sets		Perturbation Parameter τ									
		0.1	0.2	0.3	0.4	0.5	0.6	0.7	0.8	0.9	1.0
FGSM	$X^{\prime }$	0.4934	0.6430	0.7268	0.7910	0.8319	0.8594	0.8730	0.8756	0.8757	0.8805
	$X^{*}$	**0.4935**	**0.6443**	**0.7335**	**0.7980**	**0.8420**	**0.8697**	**0.8821**	**0.8829**	**0.8850**	**0.8907**
BIM	$X^{\prime }$	0.7388	0.8825	0.9135	0.9353	0.9438	0.9479	0.9532	0.9568	0.9604	0.9621
	$X^{*}$	**0.7389**	0.8825	0.9135	0.9353	0.9438	0.9479	0.9532	**0.9569**	**0.9605**	**0.9622**
PGD	$X^{\prime }$	0.5700	0.7795	0.8682	0.9038	0.9224	0.9414	0.9529	0.9558	0.9568	0.9577
	$X^{*}$	0.5700	0.7795	**0.8683**	**0.9039**	**0.9225**	**0.9416**	0.9529	0.9558	0.9568	0.9577
MI-FGSM	$X^{\prime }$	0.5686	0.7844	0.8781	0.9200	0.9412	0.9513	0.9551	0.9568	0.9583	0.9589
	$X^{*}$	0.5686	0.7844	**0.8782**	**0.9202**	**0.9413**	0.9513	0.9551	**0.9569**	0.9583	0.9589
DeepFool	$X^{\prime }$	0.8808	0.9458	0.9698	0.9811	0.9875	0.9914	0.9931	0.9944	0.9954	0.9959
	$X^{*}$	**0.8962**	**0.9514**	**0.9729**	**0.9832**	**0.9893**	**0.9930**	**0.9945**	**0.9955**	**0.9963**	**0.9968**
BS-FGM	$X^{\prime }$	0.7812	0.9019	0.9596	0.9729	0.9780	0.9813	0.9841	0.9855	0.9865	0.9873
	$X^{*}$	**0.7813**	**0.9022**	**0.9602**	**0.9734**	**0.9789**	**0.9821**	**0.9849**	**0.9864**	**0.9873**	**0.9883**

**Table 3:** ASRs of adversarial ($X^{\prime }$) and optimized ($X^{*}$) example sets generated under ResNet model.

Example Sets		Perturbation Parameter τ									
		0.1	0.2	0.3	0.4	0.5	0.6	0.7	0.8	0.9	1.0
FGSM	$X^{\prime }$	0.4718	0.6103	0.7306	0.7761	0.7922	0.7960	0.7993	0.8037	0.8100	0.8160
	$X^{*}$	**0.4744**	**0.6264**	**0.7563**	**0.7970**	**0.8103**	**0.8175**	**0.8235**	**0.8301**	**0.8393**	**0.8450**
BIM	$X^{\prime }$	0.6542	0.7143	0.7634	0.8058	0.8330	0.8589	0.8812	0.8954	0.9030	0.9064
	$X^{*}$	**0.6543**	**0.7145**	**0.7638**	**0.8062**	**0.8335**	**0.8590**	**0.8814**	**0.8956**	**0.9033**	**0.9065**
PGD	$X^{\prime }$	0.5875	0.6879	0.7502	0.8053	0.8465	0.8718	0.8884	0.8973	0.9030	0.9072
	$X^{*}$	**0.5876**	**0.6880**	**0.7505**	**0.8060**	**0.8469**	**0.8722**	**0.8890**	**0.8977**	**0.9033**	**0.9073**
MI-FGSM	$X^{\prime }$	0.5894	0.7165	0.7918	0.8333	0.8629	0.8812	0.8897	0.8925	0.8953	0.8982
	$X^{*}$	**0.5895**	**0.7167**	**0.7923**	**0.8336**	**0.8632**	**0.8814**	**0.8898**	**0.8927**	**0.8955**	**0.8984**
DeepFool	$X^{\prime }$	0.8256	0.9587	0.9801	0.9868	0.9897	0.9915	0.9926	0.9933	0.9938	0.9943
	$X^{*}$	**0.8466**	**0.9649**	**0.9833**	**0.9890**	**0.9915**	**0.9933**	**0.9942**	**0.9947**	**0.9953**	**0.9956**
BS-FGM	$X^{\prime }$	0.8313	0.9188	0.9454	0.9549	0.9600	0.9635	0.9678	0.9700	0.9708	0.9714
	$X^{*}$	**0.8325**	**0.9204**	**0.9471**	**0.9560**	**0.9607**	**0.9643**	**0.9682**	**0.9702**	**0.9710**	**0.9718**

## Data Availability

The data that support the findings of this study are available in DEEPSIG DATASET: RADIOML2018.01A at https://www.deepsig.ai/datasets, accessed on 14 May 2024.
